# Cultivating grit among college students: a comparative study between traditional physical education and outdoor education

**DOI:** 10.3389/fpsyg.2025.1485208

**Published:** 2025-10-20

**Authors:** Lun Li, ZuWang Chu, BoBo Zong, ZhengYang Zeng, YuHao Zeng, Yun Zhou

**Affiliations:** ^1^School of Physical Education, China University of Geosciences, Wuhan, China; ^2^School of Public Administration, China University of Geosciences, Wuhan, China

**Keywords:** traditional physical education, outdoor education, college students, grit, comparative study

## Abstract

**Objective:**

This study aims to address the research gap in comparing the effects of traditional physical education and outdoor education on cultivating grit among college students. Through large-scale empirical analysis, it examines the differential benefits of these two approaches, providing a critical evidence base for developing targeted psychological quality cultivation systems in higher education institutions.

**Methods:**

Comparative analysis method was used on participants consisting of 1,247 college students (traditional physical education: *n* = 809; outdoor education: *n* = 438). The original grit scale (Grit-O) was used to measure the grit quality of university students from seven universities in China before and after 16–18 weeks of both traditional physical education classes and outdoor education classes. The Mann–Whitney U test and the Wilcoxon signed-rank test were used to analyze the overall grit scores, persistence effort, and interest consistency among the participants.

**Results:**

Pre-test interest scores of college students in traditional physical education classes were significantly lower than the post-test scores. The differences in total resilience scores, persistence effort, and interest consistency among college students before and after traditional physical education classes and outdoor education classes were statistically significant. There was a significant difference in the total score of grit and persistence effort between male and female college students in traditional physical education course. The total score of grit, persistence effort, and interest consistency before and after outdoor education course were significantly improved. However, male college students showed no significant improvement in their perseverance effort through outdoor education classes.

**Conclusion:**

The findings demonstrate that both traditional physical education and outdoor education positively impact on grit of college students, with outdoor education being more effective than traditional physical education in cultivating grit among college students. However, to effectively enhance resilience of college students, traditional physical education need to focus on improving the interest consistency dimension, with a particularly emphasis on increasing persistence and effort dimension within outdoor education for male students.

## 1 Introduction

Many studies have demonstrated that college students experience increased pressure in their academics, interpersonal relationships, and career planning, which raises concerns about their grit quality (Buchanan and Cerniglia, 2020; Eisenberg et al., 2009). Therefore, in addition to imparting professional knowledge to students during their university education, it is essential to cultivate their grit quality to equip them with skills to effectively cope with social pressures post-graduation.

The grit concept was first investigated systematically by Duckworth et al. who defined it as perseverance and passion for long-term goals (2007). Although the concept of grit has been subject to academic debate since its introduction—including concerns regarding its conceptual overlap with conscientiousness (Credé et al., 2017), its cross-cultural measurement applicability (Dijksma et al., 2022), and the context-specific nature of its original theoretical development (Lee et al., 2023)—the two-dimensional framework comprising ‘perseverance of effort’ and ‘consistency of interest’, as proposed by Duckworth et al., remains the dominant paradigm in operationalizing grit in empirical research. This study adopts this operational definition for several reasons: first, the model offers strong measurability and comparability across populations, facilitating the quantitative assessment of intervention effects; second, it allows this research to maintain a common dialogue with the extensive existing literature; and third, the two-dimensional structure provides a refined analytical lens for comparing the differential effects of various educational pathways.

Grit is an important psychological trait during an individual’s development, particularly in the growth and success of college students (Duckworth, 2016). Grit has been demonstrated to be significantly associated with educational attainment (Duckworth et al., 2007), age (Eskreis-Winkler et al., 2014), growth mindset (Tucker-Drob et al., 2016; Yeager et al., 2016), optimistic explanatory style (Robertson-Kraft and Duckworth, 2014), self-control (Duckworth and Gross, 2014), delay of gratification (Meriac et al., 2015), goal commitment (Hill et al., 2016), self-efficacy (Jordan et al., 2015), teacher-student and parent–child relationships (Datu, 2017), and physical performance (Buller, 2012). Notably, in 2 of 4 studies reported in Martin, the authors report that for individuals who have jobs that require them to be physically active, there is no significant association between physical activity and grit (Martin et al., 2023). However, grit is not significantly associated with intelligence (Duckworth et al., 2007). Moreover, grit is dynamic concept (Rutberg et al., 2020) that was developed within a specific context (Lee et al., 2023). Therefore, its conceptualization remains debatable with its application in different measurement contexts, such as sports psychology, considered limited (Credé et al., 2017; Credé and Tynan, 2021; Dijksma et al., 2022). Further research is required to determine effective strategies for cultivating grit at both individual and collective levels (Schimschal et al., 2021).

Existing research on the relationship between physical activity and grit is fragmented and lacks systematic integration and comparison. Studies have found that grit among undergraduate students is positively correlated with participation in family and leisure time sports activities (Daniels et al., 2023). Dunston et al. (2022) suggests that the intensity of physical activities among college students substantially influence psychosocial determinants, such as grit and resilience, associated with success in students. Research by Frontini among undergraduate students majoring in sports revealed significant differences in enthusiasm, grit, and mentality between genders (Roberta et al., 2021). Deitchler and Biechler (2022) found that male collegiate athletes exhibited significantly higher grit scores than their female counterparts. Additionally, Gilchrist et al. (2017) posits that creating opportunities for college students to experience self-pride through sports activities can help in cultivating grit. As with other personality traits, grit is not fixed and may change across the lifespan as a result of life experiences and contextual influences (Nothnagle and Knoester, 2022). Additionally, higher educational attainment has been positivelyassociated with both grit (Lam and Zhou, 2019) and PA participation (Gerovasili et al., 2015). Grit was positively associated with physical activity (Daniels et al., 2023).

Meanwhile, outdoor education, as another form of experiential learning, has also been shown to enhance grit, though its mechanisms of action may differ from those of traditional physical education. Moreover, research indicates that outdoor educational activities such as hiking and rock climbing significantly improve students’ self-awareness and grit (Hattie et al., 1997). Students facing outdoor challenges need to overcome both physical and psychological discomfort, which enhance their resilience and adaptability (Beames and Brown, 2014). Additionally, outdoor education promotes development of teamwork and leadership skills that effectively enhance grit (Boss, 1999). Common knowledge implies that individuals engaging in outdoor sports and especially in regular and extreme mountaineering are exceptionally healthy and hardened (Habelt et al., 2023). Existing research demonstrates that mountaineering can enhance grit levels among college students (Li et al., 2025).

However, significant gaps remain in the current literature: first, most studies focus on a single type of activity and lack direct comparisons between traditional physical education and outdoor education; second, few studies explore the differential effects of these two educational approaches on distinct dimensions of grit (perseverance of effort vs. consistency of interest); and third, there is a scarcity of large-sample empirical evidence to determine which approach is more effective.

In summary, given the current lack of rigorous empirical evidence directly comparing the effectiveness of these two widely adopted educational approaches, this study carries significant practical and theoretical necessity. Our large-sample, head-to-head comparison moves beyond theoretical speculation to determine which approach is more effective in fostering grit and to analyze dimensional differences in their outcomes. This directly responds to Schimschal et al. (2021)‘s call for identifying best practices in grit cultivation. Furthermore, the findings inform the development of evidence-based strategies to enhance grit within university physical education programs, ultimately helping students better cope with future challenges.

The primary aim to investigate the differential impacts of traditional physical education versus outdoor education on cultivating grit among college students, framed within the dual context of Duckworth’s grit theory and the challenge-based learning paradigm, with specific hypotheses proposing that (1) both educational approaches would significantly enhance grit scores but through distinct psychological mechanisms (traditional PE through interest consistency development and outdoor education through perseverance cultivation), (2) outdoor education would demonstrate superior efficacy in overall grit enhancement particularly in persistence effort, and (3) gender differences would emerge more prominently in traditional PE settings.

## 2 Method

### 2.1 Curriculum

Students across seven universities were selected for the spring semester of 2024 (February 26 to June 28, 2024) to participate in either the traditional physical education courses or the outdoor education courses. Both course lasted for 16–18 weeks, with one class session per week and each session lasting for 90 min including a 5-min break midway through. Each session consisted of one instructor and 20–45 students. Traditional physical education courses included basketball, football, volleyball, badminton, aerobics, ballroom dancing, slow pitch softball, taekwondo, and tai chi. Contrarily, outdoor education courses included wilderness survival experiences, Outward Bound movement, camping, rock climbing, and mountain climbing.

### 2.2 Participants

As participants self-selected into traditional physical education or outdoor education courses prior to the study, this investigation adopts a quasi-experimental design comparing naturally occurring groups. Participants were recruited using online questionnaires, without being clearly informed of the quasi-experimental details. Participation was voluntary. The inclusion criteria were as follows: (1) Individuals aged between 18–25 years old. (2) A college student enrolled at a university. (3) A physically healthy student capable of independently completing one semester of coursework and practical activities, with at most one absence. (4) Ability to speak native Chinese language with fluency in Mandarin. (5) Full participation in classroom teaching activities throughout semester. The exclusion criteria were as follows: (1) Presence of serious physical or mental illness, or a current condition, such as pregnancy, that renders participation in coursework unsuitable. (2) Experiencing significant life events or strong emotional fluctuations within the semester. (3) Inability to complete all course activities and tests.

A total of 1,353 college students participated in the study. Of these, 1,247 students (Physical education course: *n* = 809, including 405 males and 404 females; Outdoor education course: *n* = 438, including 281 males and 157 females) completed all tests. The remaining 106 students withdrew from the study from the study for various reasons: 31 students withdrew midway due to course schedule conflicts; 46 students missed 2 or more class sessions; 24 students experienced serious health challenges; and 5 students withdrew without any particular reason.

### 2.3 Procedure

Convenience sampling was used in the distribution of questionnaires and data collection. The questionnaires were developed using the Wenjuanxing platform. Participants logged into the platform by scanning a QR code using their smartphones, and then independently completed the questionnaire items within the first 10 min of the first class (February 26th to March 1st) and the last 10 min of the final class (June 24th to June 28th) of the semester. The QR codes were sent to the course instructors in advance to publish and avail to the students during the scheduled testing dates. The questionnaire included the Grit - O and demographic questions on name, age, gender, and course title. Additional questions addressed a participant’s injury and mental health statuses. An information sheet prefaced the questionnaire and acted as an informed consent form.

While participant names were collected for within-subject matching purposes, all personally identifiable information was immediately anonymized following data collection. The name-matching procedure was conducted solely to ensure accurate pre-post test pairing, and no identifiable information is included in the published dataset. Informed consents were obtained from all participants, and participant withdrawal from the study was allowed without any responsibility. The study was been approved by the Ethics Committee of China University of Geosciences in Wuhan.

### 2.4 Instrument

The Grit-O consists of 12 items rated on a 5-point Likert scale (where 1 = not at all like me, 5 = very much like me). The scale has two dimensions: interest consistency and persistence effort (Duckworth et al., 2007). Examples of items assessed were consistency in long - term interests (e.g., “I find it difficult to focus on projects that take several months to complete”) and the ability to persevere in adversity (e.g., “I overcome an important challenge by overcoming setbacks”). The internal consistency coefficient of Grit-O scale is strong with a value of 0.85. In 2009, Duckworth and Quinn revised the Grit-O by removing four items, resulting in the Short Grit Scale (Grit - S) comprising eight items (Duckworth and Quinn, 2009). In 2018, Zhang Nan simultaneously revised the Chinese versions of Grit - O and Grit - S, and then performed reliability and validity tests among Chinese college students (Nan, 2018). The results showed that Grit - O had a strong construct validity and a better performance compared to Grit-S making it a reliable for measuring grit levels among Chinese college students.

### 2.5 Statistical analysis

Statistical analyses were performed at a significance level (*α*) of 0.05(two-tailed) using SPSS software (version 27 and IBM Corp model). Data were presented as mean ± standard deviation (SD), median (interquartile range [IQR], or n [%]). Kolmogorov - Smirnov test was used to assess the normality of distribution the sample data. The assumption of homogeneity of variance was tested using Levene’s test. Wilcoxon signed-rank test was used to assess the differences in resilience quality scores before and after traditional physical education and outdoor education courses among the participants. The non parametric Mann - Whitney U test was used to investigate the inter group differences in resilience qualities between traditional sports and outdoor education students. To mitigate the influence of sample size disparity on the interpretation of results, effect sizes (r, Rosenthal’s correlation coefficient) were calculated and reported alongside p - values for both Wilcoxon signed - rank and Mann - Whitney U tests. Effect sizes were interpreted according to the standards proposed by Cafri et al. (2010). Multivariate linear regression models were employed to mitigate Type I error inflation from multiple comparison testing while systematically examining intervariable relationships.

## 3 Results

Normality tests were performed for the following variables: overall grit score, effort score, and interest score both pre and post participation in the traditional physical education course, as well as for outdoor educational course. Normality tests were also conducted for the differences in the overall grit scores, effort scores, and interest scores pre and post participation for both courses (see details in Table 1).

**Table 1:** Normality test analysis results of the overall grit score, interest consistency and persistence effort dimensions of college students in traditional physical education courses and outdoor education courses.

Scales	Variable	Mean ± SD		Skewness		Peakness		Kolmogorov–Smirnov test			
								*D*		*p*	
		PE	OE	PE	OE	PE	OE	PE	OE	PE	OE
Overall grit score	pre	38.14 ± 5.734	38.53 ± 6.064	0.271	0.543	0.454	0.369	0.068	0.096	0.000***	0.000***
	post	39.14 ± 6.080	41.40 ± 6.255	0.387	0.561	0.679	0.324	0.094	0.104	0.000***	0.000***
	pre-post	1.00 ± 0.179	2.86 ± 0.13	0.363	2.103	6.229	23.353	0.089	0.334	0.000***	0.000***
Persistence effort	pre	19.18 ± 4.008	19.51 ± 4.144	−0.062	0.050	0.397	−0.027	0.062	0.085	0.000***	0.000***
	post	20.90 ± 4.505	21.58 ± 4.520	−0.276	−0.120	0.307	0.133	0.067	0.068	0.000***	0.000***
	pre-post	1.72 ± 0.138	2.07 ± 0.100	0.211	0.612	4.430	12.616	0.117	0.344	0.000***	0.000***
Interest consistency	pre	18.96 ± 3.976	19.03 ± 4.501	−0.268	−0.364	0.462	0.520	0.088	0.079	0.000***	0.000***
	post	18.24 ± 4.406	19.82 ± 4.385	−0.138	−0.212	0.747	0.221	0.096	0.069	0.000***	0.000***
	pre-post	−0.71 ± 0.157	0.80 ± 0.123	−0.178	1.568	3.225	19.982	0.099	0.389	0.000***	0.000***

PE: traditional physical education; OE: outdoor education; pre-post: Subtract the difference between post test and pre test.

*p < 0.05; **p < 0.01; ***p < 0.001.

For all outcome measures and between-group comparisons, homogeneity of variance was assessed using Levene’s test (results presented in Tables 2, 3).

**Table 2:** Homogeneity of variance test results for pre-test, post-test, and pre-post difference scores of the course grit total, perseverance of effort, and consistency of interest dimensions among college students in traditional physical education versus outdoor education courses.

Variable	Levene’s Test	df₁	df_2_	*p*
Pre course grit total	1.932	1	1,245	0.165
Pre persistence effort score	1.705	1	1,245	0.192
Pre interest consistency score	5.993	1	1,245	0.015*
Post course grit total	0.386	1	1,245	0.535
Post persistence effort score	0.301	1	1,245	0.583
Post interest consistency score	0.747	1	1,245	0.387
Difference in total grit score before and after	151.897	1	1,245	0.000***
Difference in persistence effort before and after	141.974	1	1,245	0.000***
Difference in interest consistency before and after	154.883	1	1,245	0.000***

*p < 0.05; **p < 0.01; ***p < 0.001.

**Table 3:** Between-group comparison results of pre-test, post-test, and pre-post difference scores for the course grit total, perseverance of effort, and consistency of interest dimensions among college students in traditional physical education versus outdoor education courses.

Grouping	*n*	Pre course grit total	Pre persistence effort score	Pre interest consistency score	Post course grit total	Post persistence effort score	Post interest consistency score	Difference in total grit score before and after	Difference in persistence effort before and after	Difference in interest consistency before and after
PE	809	38.14 ± 5.73	19.18 ± 4.01	18.96 [19.22]	39.14 ± 6.08	20.90 ± 4.51	18.24 ± 4.41	1.00 [−2.4]	1.72 [0.4]	−0.71 [−3.2]
OE	438	38.53 ± 6.06	19.51 ± 4.14	19.03 [16.22]	41.40 ± 6.26	21.58 ± 4.52	19.82 ± 4.39	2.87 [3.3]	2.07 [2.2]	0.80 [1.1]
t (or Z)		*t* = 1.13	*t* = 1.36	*Z* = −0.375	*t* = 6.19	*t* = 2.54	*t* = 6.05	*Z* = −0.9.62	*Z* = −0.375	*Z* = 0.8.73
*p*		0.16	0.19	0.71	0.54	0.58	0.39	0.00***	0.00***	0.00***

PE: traditional physical education; OE: outdoor education.

Normally distributed continuous data are presented as mean ± standard deviation (x̄ ± SD); non-normally distributed continuous data are expressed as median (25th percentile, 75th percentile) M [P25, P75].

*p < 0.05; **p < 0.01; ***p < 0.001.

With sample sizes in this study being greater than 50, the K-S test was used to test for the normality of the data (Razali and Wah, 2011). As demonstrated in Table 1, the analysis revealed that all the variables exhibited significant differences (*p* < 0.05) for both course pre and post participation, resulting in the rejection of the null hypothesis of normal data distribution. Due to non-normality of the data distribution, Wilcoxon signed-rank test with paired samples was used (2011). The results are shown in Table 4.

**Table 4:** Comparison of college students’ overall grit score, interest consistency, and persistence effort after traditional physical education courses and outdoor education courses.

Grouping	Median pairing M (P_25_, P_75_)	Median difference (pre-post)	Wilcoxon test		
			*z*	*p*	*r*
Pre course grit total score of PE	38.000 (34.0, 42.0)	0.000	−6.254	0.000***	0.220
Post course grit total score of PE	38.000 (35.0, 43.0)				
Pre persistence effort score of PE	19.000 (16.0, 22.0)	−2.000	−13.179	0.000***	0.463
Post persistence effort score of PE	21.000 (18.0, 24.0)				
Pre interest consistency score of PE	19.000 (17.0, 22.0)	1.000	−4.569	0.000***	0.161
Post interest consistency score of PE	18.000 (16.0, 21.0)				
Pre course grit total score of OE	38.000 (34.0, 42.0)	−3.000	−16.626	0.000***	0.794
Post course grit total score of OE	41.000 (37.0, 45.0)				
Pre persistence effort score of OE	19.000 (16.0, 22.0)	−2.000	−16.581	0.000***	0.792
Post persistence effort score of OE	21.000 (18.0, 24.8)				
Pre interest consistency score of OE	19.000 (16.3, 22.0)	−1.000	−12.112	0.000***	0.579
Post interest consistency score of OE	20.000 (17.0, 23.0)				

PE: traditional physical education; OE: outdoor education; pre-post: Subtract the difference between post test and pre test.

*p < 0.05; **p < 0.01; ***p < 0.001.

The results showed that there were significant improvements in resilience scores after both courses. The test showed significant increases in the: effort score after traditional physical education (*Z* = −13.179, *p* < 0.05); total score (*Z* = −16.626, *p* < 0.05), effort score (*Z* = −16.581, *p* < 0.05), and interest score (*Z* = −12.112, *p* < 0.05) after outdoor education course, indicating that after the coursework, the resilience, effort, and interest dimensions of college students were significantly improved. The median total resilience score after traditional physical education courses was 38 which was significant (*Z* = −6.254, *p* < 0.05), indicating that the resilience score of college students has significantly improved. The median interest scores of college students after traditional physical education courses was significantly lower (*Z* = −4.569, *p* < 0.05), indicating a significant decrease in interest scores after traditional physical education courses. Although the PE group yielded large z-values, the effect sizes were relatively small due to the larger sample size (*N* = 809). All effect sizes in the OE group (0.579–0.794) were substantially larger than those in the PE group (0.161–0.463), indicating stronger practical effects of outdoor education.

A Mann–Whitney U test was conducted to determine the differences between the two groups, and the results are shown in Table 5. The results showed that there was a statistically significant differences in the total score of resilience (*Z* = −9.617, *p* < 0.05), effort (*Z* = −3.750, *p* < 0.05), and interest (*Z* = −8.733, *p* < 0.05) before and after both traditional physical education and outdoor education courses, indicating a significant improvement in all dimensions of resilience quality after the courses. Although the difference in the perseverance dimension was statistically significant, the effect size was small (*r* = 0.106), suggesting limited practical importance. Differences in total grit scores and the interest dimension were more substantial.

**Table 5:** Non parametric test analysis of the differences in the overall grit score, interest consistency, and persistence effort before and after the completion of traditional physical education courses and outdoor education courses.

Classification	Median pairing M (P_25_, P_75_)		Mann Whitney test		
	PE (*n* = 809)	OE (*n* = 438)	z	*p*	*r*
Difference in total grit score before and after	1.000 (−2.0, 4.0)	3.000 (3.0, 3.0)	−9.617	0.000***	0.272
Difference in persistence effort before and after	1.000 (0.0, 4.0)	2.000 (2.0, 2.0)	−3.750	0.000***	0.106
Difference in interest consistency before and after	−1.000 (−3.0, 2.0)	1.000 (1.0, 1.0)	−8.733	0.000***	0.247

PE: traditional physical education; OE: outdoor education.

*p < 0.05; **p < 0.01; ***p < 0.001.

A paired-sample Wilcoxon signed-rank test (Razali and Wah, 2011) was conducted and the results are shown in Table 6. The results showed that the median score of college girls before and after traditional physical education courses was 38, which indicated a significant improvement in the total score of resilience qualities among female college students before and after traditional physical education courses (*Z* = −3.440, *p* < 0.05). Moreover, there were significant differences between male and female college students in the total score, effort score, and interest score before and after traditional physical education courses, indicating significant improvements. Although there is a significant difference in interest scores between male and female college students in traditional physical education courses, with a median difference of 1.000, the interest scores of college students in traditional physical education courses significantly decreased. All effect sizes in the OE group (0.469–0.885) were considerably larger than those in the PE group (0.137–0.479). Gender differences were notable within OE, where females showed higher effect sizes than males, particularly in the interest dimension (0.885 vs. 0.469). Dimension-wise, improvements in perseverance were most pronounced in both groups (PE: 0.446–0.479; OE: 0.769–0.805).

**Table 6:** Comparison of the overall grit score, interest consistency, and persistence effort between male and female college students after traditional physical education courses and outdoor education courses.

Grouping	Gender	Median pairing M (P_25_, P_75_)		Median difference (pre-post)	Wilcoxon test		
		pre	post		*z*	*p*	*r*
The overall grit score of PE	Male (405)	37.000 (34.0, 42.0)	38.000 (35.0, 43.0)	−1.000	−5.372	0.000***	0.267
	Female (404)	38.000 (34.0, 42.0)	38.000 (35.0, 43.0)	0.000	−3.440	0.001**	0.171
Persistence effort of PE	Male (405)	19.000 (16.0, 22.0)	21.000 (18.0, 24.0)	−2.000	−9.633	0.000***	0.479
	Female (404)	19.000 (16.0, 22.0)	21.000 (18.0, 23.0)	−2.000	−8.973	0.000***	0.446
Interest consistency of PE	Male (405)	19.000 (16.5, 21.0)	18.000 (16.0, 21.0)	1.000	−2.758	0.006**	0.137
	Female (404)	19.000 (17.0, 22.0)	18.000 (16.0, 21.0)	1.000	−3.728	0.000***	0.185
The overall grit score of OE	Male (281)	38.000 (34.0, 43.0)	41.000 (37.0, 46.0)	−3.000	−12.524	0.000***	0.747
	Female (157)	37.000 (34.0, 41.5)	40.000 (37.0, 43.5)	−3.000	−10.470	0.000***	0.835
Persistence effort of OE	Male (281)	20.000 (17.0, 23.0)	22.000 (18.5, 25.0)	−2.000	−12.891	0.000***	0.769
	Female (157)	18.000 (16.0, 21.0)	21.000 (18.0, 23.0)	−3.000	−10.082	0.000***	0.805
Interest consistency of OE	Male (281)	19.000 (16.0, 23.0)	20.000 (17.0, 23.0)	−1.000	−7.860	0.000***	0.469
	Female (157)	18.000 (17.0, 21.0)	19.000 (18.0, 22.0)	−1.000	−11.093	0.000***	0.885

PE: traditional physical education; OE: outdoor education; pre-post: Subtract the difference between post test and pre test.

*p < 0.05; **p < 0.01; ***p < 0.001.

A Mann–Whitney U test was conducted to determine the difference in scores between male and female college students before and after physical education and outdoor courses. The results are shown in Table 7. The results showed that there was a significant improvement in the total score (*Z* = −5.911, *p* < 0.05) and interest score (*Z* = −5.904, *p* < 0.05) of male college students before and after physical education and outdoor courses. However, no significant difference was observed in the effort score (*Z* = −5.911, *p* = 0.124). There were significant differences in the total score, effort, and interest of female college students before and after physical education and outdoor courses. Effect sizes indicate that outdoor education produced stronger improvements in total grit, interest, and perseverance among female students.

**Table 7:** Comparison of the differences in the overall grit score, interest consistency and persistence effort between male and female college students after traditional physical education courses and outdoor education courses.

Classification	Gender	Median pairing M (P_25_, P_75_)		Mann–Whitney test		
		PE (Male = 405; Female = 404)	OE(Male = 281; Female = 157)	*z*	*p*	*r*
Difference in the overall grit score before and after	Male	1.000 (−1.0, 4.0)	3.000 (2.0, 3.0)	−5.911	0.000***	0.226
	Female	1.000 (−2.0, 3.0)	3.000 (3.0, 3.0)	−7.660	0.000***	0.323
Difference in the persistence effort before and after	Male	2.000 (0.0, 4.0)	2.000 (2.0, 2.0)	−1.538	0.124	0.059
	Female	1.000 (0.0, 3.0)	2.000 (2.0, 2.0)	−3.670	0.000***	0.155
Difference in the interest consistency before and after	Male	0.000 (−3.0, 2.0)	1.000 (1.0, 1.0)	−5.904	0.000***	0.225
	Female	−1.000 (−3.0, 2.0)	1.000 (1.0, 1.0)	−6.499	0.000***	0.274

PE: traditional physical education; OE: outdoor education.

*p < 0.05; **p < 0.01; ***p < 0.001.

Using multivariate linear regression with only existing variables (group, sex, pre−/post-test scores) and no additional data, results remained consistent with prior statistical analyses (see Table 8).

**Table 8:** Multiple linear regression results for the course grit total, perseverance of effort, and consistency of interest among college students in traditional physical education versus outdoor education courses.

Model		Unstandardized coefficient		Standardized coefficient	*t*	*p*
		Beta	Standard error	Beta		
Post course grit total	Intercept	47.787	1.887		25.323	0.000***
	Grouping	−4.247	1.113	−0.325	−3.816	0.000***
	Gender	−3.051	1.295	−0.244	−2.355	0.019*
	Grouping × Gender	1.475	0.748	0.274	1.973	0.049
Post persistence effort score	Intercept	24.678	1.385		17.819	0.000***
	Grouping	−1.567	0.817	−0.166	−1.919	0.055
	Gender	−1.827	0.951	−0.201	−1.922	0.055
	Grouping × Gender	0.698	0.549	0.179	1.273	0.203
Post interest consistency score	Intercept	23.109	1.354		17.066	0.000***
	Grouping	−2.680	0.799	−0.287	−3.356	0.001**
	Gender	−1.224	0.930	−0.137	−1.317	0.188
	Grouping × Gender	0.777	0.536	0.202	1.448	0.148
Difference in total grit score before and after	Intercept	3.358	1.360		2.469	0.014*
	Grouping	−0.873	0.802	−0.093	−1.088	0.277
	Gender	0.967	0.934	0.107	1.036	0.301
	Grouping × Gender	−0.687	0.539	−0.177	−1.276	0.202
Difference in persistence effort before and after	Intercept	1.977	1.043		1.895	0.058
	Grouping	0.048	0.615	0.007	0.078	0.938
	Gender	0.302	0.716	0.044	0.422	0.673
	Grouping × Gender	−0.270	0.413	−0.092	−0.654	0.513
Difference in interest consistency before and after	Intercept	1.382	1.201		1.150	0.250
	Grouping	−0.921	0.708	−0.111	−1.300	0.194
	Gender	0.664	0.825	0.083	0.806	0.421
	Grouping × Gender	−0.417	0.476	−0.122	−0.877	0.381

*p < 0.05; **p < 0.01; ***p < 0.001.

## 4 Discussion

The results from this study indicate that the resilience scores of college students before and after traditional physical education and outdoor education are not normally distributed. College students who undertook these courses for 16–18 weeks have shown significant improvement in all aspects except for a significant decrease in interest within traditional physical education. College students who participate in outdoor education courses show significantly higher improvement in their overall resilience, effort, and interest dimensions compared to traditional physical education courses. The improvement in the effort dimension of college male students through outdoor education courses did not differ significantly from that observed in the traditional physical education.

Building on these findings, the discussion will proceed along three key dimensions: first, elucidating the core theoretical contributions of this study and its dialogue with existing research; second, analyzing potential mechanisms underlying the decline in student interest within traditional physical education courses; and finally, exploring the enhancing effect of outdoor education on perseverance and its gender-specific patterns, along with proposing practical educational recommendations.

One of the main contributions of this study is the investigation that outdoor education significantly improves resilience of college students in terms of overall score, effort, and interest. This is an area that has been unexplored. Previous studies have explored the relationship between physical activity and resilience qualities in college students (Fitzgerald and McEwan, 2020). A study by Crawford and Godbey (1987) provides insights into the impact of leisure activities on individual psychological traits, indirectly supporting the notion that physical activity enhances resilience qualities (Crawford and Godbey, 1987). The findings of this study not only confirm the general benefits of physical activity for grit development but also reveal significant differences in effectiveness across types of physical activities, providing new empirical evidence for understanding how different athletic contexts differentially shape psychological traits.

Physical activity has a beneficial impact on the resilience of college students (Daniels et al., 2023; Yu et al., 2024). Our research confirms that both traditional physical education and outdoor education have significant impact on the overall resilience and perseverance of college students. However, we found that both male and female college students showed a significant decrease in interest scores before and after traditional physical education courses.

Regarding the observed decline in student interest within traditional physical education courses, we believe this finding carries important theoretical and practical implications. Research has found that there are various factors that affect interest of college students in physical education courses, including course content, instructor’s teaching methods, peer influence, and university sports facilities (Xiao, 2019). For example, modern teaching methods such as interactive teaching and gamified learning effectively enhance participation interest of students (Mian, 2018). We propose two possible reasons for the decrease in interest consistency: first, the relatively fixed teaching environment in traditional physical education courses compared to the varied experiences in outdoor education cause reduced novelty and engagement over time. This reason is consistent with the findings of Schunk et al. (2008); second, as students’ initial expectations for novelty and social interaction in traditional physical education shift toward a desire for more challenging and diverse activities, their interest may fade (Hidi and Renninger, 2006). It is noteworthy that outdoor education demonstrated a marked improvement in the dimension of interest, in sharp contrast to traditional physical education. This discrepancy can be explained through several key mechanisms. First, environmental novelty plays a critical role in sustaining learning interest. Research by Anggereini and Yelianti (2023) indicates that the diversity and unpredictability of natural environments stimulate exploratory behavior and maintain attention - an effect difficult to achieve in repetitive indoor settings (Anggereini and Yelianti, 2023). Second, Self-Determination Theory provides a theoretical framework for this phenomenon. Ryan and Deci (2000) emphasized that the satisfaction of three basic psychological needs - autonomy, competence, and relatedness—is essential for maintaining intrinsic motivation (Ryan and Deci, 2000). Outdoor education better fulfills these needs by offering more opportunities for autonomous choice, appropriately challenging tasks, and rich social interactions. Furthermore, differences in curriculum design are also a significant factor. Patall et al. (2008) found that outdoor education activities often incorporate more diverse and exploratory content designs, such as problem-solving and team-based tasks, which provide continuous cognitive stimulation and a sense of achievement(Patall et al., 2008). Lastly, differences in teaching style cannot be overlooked. Research by Wen-Ge (2007) suggests that outdoor educators tend to adopt a more democratic and facilitative teaching style, reducing the potential pressure associated with the authoritarian instruction often found in traditional physical education (Wen-Ge, 2007). Therefore, the observed differences in interest between traditional physical education and outdoor education reflect not only the short-term effects of environmental novelty but also systematic differences in curricular design, the fulfillment of psychological needs, and modes of social interaction.

With respect to the perseverance dimension, this study identified interesting gender-based patterns. Previous studies have confirmed the effectiveness of outdoor courses in enhancing effort of college students, mainly through increased participation (Becker and Wiggins, 2016), challenge (Fletcher et al., 2020), social support (Côté and Fraser-Thomas, 2007), and mental health awareness (Barton and Pretty, 2010). Outdoor education provides abundant practical opportunities that can stimulate students’ interest and participation (Becker et al., 2017). Similarly, traditional sports can enhance students’ effort through diverse activities and teamwork spirit (Smith and Johnson, 2019). These findings indicate that the effectiveness of these courses depends on the teaching methods and student attitudes. Although our research did not find a significant difference between traditional physical education and outdoor education in improving the effort of college students, outdoor education exhibits significant effects in other areas such as social skills, teamwork, and mental health (Williams et al., 2020). Considering diverse student backgrounds and personal interests, educators should consider diverse teaching methods when designing courses to meet these needs (Parker, 2021). For example, increasing the diversity of outdoor activities or introducing more exploratory learning elements into traditional physical education courses may further stimulate students’ learning motivation and effort.

Particularly noteworthy is the finding that male and female university students showed limited improvement in perseverance within both traditional and outdoor education settings. Another significant finding of this study is the lack of significant improvement in the effort dimension between male students in outdoor education courses and traditional physical education courses. Conversely, there is a significant improvement in effort in outdoor courses. These findings indicate that similarity in effort dimension between male college students participating in outdoor education courses and traditional physical education courses is attributed to their competitiveness and initiative in sports activities. Research has shown that male college students are generally more inclined to participate in challenging activities, and traditional physical education courses already meet their needs for competition and achievement (Smith and Johnson, 2019). Therefore, the additional motivational effect of outdoor courses on their effort dimension is limited. In contrast, female college students showed a significant improvement in their effort dimension in outdoor courses, indicating that outdoor activities are more attractive to them. This is attributed to the social interaction and teamwork opportunities provided by outdoor courses. Research shows that outdoor courses often promote cooperation and communication among students, thereby enhancing motivation of female students to participate (Becker et al., 2017). Additionally, changes and novelty in outdoor environments may motivate female college students to be more actively engaged in learning (Williams et al., 2020). Gender differences in the effort improvement are attributed to socio-cultural factors, with outdoor activities providing female students with a more friendly environment, promoting their confidence and willingness to participate (Parker, 2021). Educators should factor in gender difference curriculum design to improve the effort dimension. The design for outdoor education courses for female students can emphasize collaboration and social elements to increase their participation and effort levels, while introduction of exploratory and teamwork activities should be considered for male students during curriculum design (Smith and Johnson, 2019).

Based on these findings, we propose the following recommendations for educational practice: Educators should account for the different needs of male and female students in curriculum design, particularly concerning the enhancement of perseverance. Outdoor programs targeting female students could place greater emphasis on cooperative and social elements to further boost their engagement and perseverance levels. Meanwhile, courses designed for male students could incorporate more inquiry-based and team-oriented activities to stimulate their interest and effort (Smith and Johnson, 2019). Considering the diverse backgrounds and interests of students, educators should adopt integrated and flexible teaching approaches to better meet varied needs (Parker, 2021). For example, increasing the diversity of outdoor activities or introducing more exploratory learning components into traditional physical education may further enhance students’ motivation and perseverance.

In summary, through a systematic comparison of the effects of traditional physical education and outdoor education on grit among university students, this study not only reveals the differential effectiveness of these two educational models in enhancing distinct dimensions of grit but also identifies the moderating role of gender in this process. These outcomes provide an important theoretical foundation and practical guidance for the reform of physical education in higher education.

## 5 Conclusion

This study shows that improvement in the overall grit score, interest consistency, and persistence effort among college students is significantly higher in outdoor education compared to traditional physical education. We propose specific evidence-based recommendations for curriculum designers to implement hybrid pedagogical models that integrate the interest-enhancing strategies from traditional physical education with the perseverance-building elements of outdoor education, with particular emphasis on gender-tailored interventions that enhance competitiveness for male students while preserving the psychologically supportive environment that fosters interest development. Future traditional physical education courses should focus in enhancing the interest of college students by diversifying the teaching environment and course content. For male students, outdoor education courses need to enhance their competitiveness and initiative to improve their resilience and effort.

### 5.1 Limitations and prospects of this study

While snowball sampling proved pragmatically necessary to access sufficient participants across both traditional physical education and outdoor education programs in multiple universities, we fully acknowledge this non-probability approach may affect the generalizability of our findings. This study did not account individual differences among students, such as professional background and psychological traits, which may have an impact on the cultivation of resilience qualities.

Future research should distinguish between various outdoor activities and identify those that are most effective in cultivating resilience in students. Additionally, further research is required to explore the comprehensive impact of traditional sports and outdoor education on psychological and social aspects, and investigate the gender-specific needs and experiences to optimize curriculum design and improve participation and effort levels of students.

## Data Availability

The raw data supporting the conclusions of this article will be made available by the authors, without undue reservation.

## References

[ref1] Anggereini E. Yelianti U. (2023). Student's responses to pro-environmental behavior-based learning and its effect on interest and critical thinking. Jurnal Pendidikan dan Pengajaran 56, 126–138. doi: 10.23887/jpp.v56i1.50166

[ref2] Barton J. Pretty J. (2010). What is the role of green space in enhancing mental well-being? J. Environ. Psychol. 30, 179–188.

[ref3] Beames S. Brown M. (2014). Enough of ronald and mickey: focusing on learning in outdoor education. J. Adventure Educ. Outdoor Learn. 14, 118–131. doi: 10.1080/14729679.2013.841096

[ref4] Becker C. Smith B. Johnson J. (2017). Outdoor education and student engagement: a review of the literature. J. Adventure Educ. Outdoor Learn. 17, 115–130.

[ref5] Becker K. Wiggins A. (2016). The impact of physical education on student engagement. J. Physical Educ. Recreation Dance 87, 36–42.

[ref6] Boss J. A. (1999). Outdoor education and the development of civic responsibility. Eric digest. Adventure Educ. (1):4.

[ref7] Buchanan E. M. Cerniglia L. (2020). The impact of academic stress on college students: a longitudinal study. J. Coll. Stud. Dev. 61, 487–501.

[ref8] Buller E. F. (2012). The relationship between grit and academic, military and physical performance at the United States military academy. Int. J. Radiation Oncol. Biol. Physics 93, 702–709. doi: 10.1016/j.ijrobp.2015.06.041

[ref9] Cafri G. Kromrey J. D. Brannick M. T. (2010). A Meta-Meta-analysis: empirical review of statistical power, type I error rates, effect sizes, and model selection of Meta-analyses published in psychology. Multivar. Behav. Res. 45, 239–270. doi: 10.1080/00273171003680187, PMID: 26760285

[ref9001] Côté J. Fraser-Thomas J. (2007). Youth involvement in sport. In Sport psychology: A Canadian perspective. (ed.) Crocker P. R. E. (Pearson Education Canada), pp. 266–294.

[ref10] Crawford D. W. Godbey G. (1987). Reconceptualizing barriers to family leisure. Leis. Sci. 9, 119–127. doi: 10.1080/01490408709512151

[ref11] Credé M. Tynan M. C. (2021). Should language acquisition researchers study “grit”? A cautionary note and some suggestions. J. Psychol. Lang. Learn. 3, 37–44. doi: 10.52598/jpll/3/2/3

[ref12] Credé M. Tynan M. C. Harms P. D. (2017). Much ado about grit: a meta-analytic synthesis of the grit literature. J. Pers. Soc. Psychol. 113, 492–511. doi: 10.1037/pspp0000102, PMID: 27845531

[ref13] Daniels B. T. Human A. E. Gallagher K. M. Howie E. K. (2023). Relationships between grit, physical activity, and academic success in university students: domains of physical activity matter. J. Am. Coll. Heal. 71, 1897–1905. doi: 10.1080/07448481.2021.1950163, PMID: 34242136

[ref14] Datu J. A. D. (2017). Sense of relatedness is linked to higher grit in a collectivist setting. Pers. Individ. Differ. 105, 135–138. doi: 10.1016/j.paid.2016.09.039

[ref15] Deitchler C. Biechler E. (2022). Differences in college athlete grit scores between sex and student year in school: 2614. Medicine & Science in sports & Exercise 54, 521. doi: 10.1249/01.mss.0000881584.02121.df

[ref16] Dijksma I. Lucas C. Stuiver M. (2022). Grit was not associated to dropout in Dutch marine recruits. Mil. Psychol. 34, 616–621. doi: 10.1080/08995605.2022.2028518, PMID: 38536348 PMC10013494

[ref17] Duckworth A. L. (2016). Grit: The power of passion and perseverance. New York: Scribner.

[ref18] Duckworth A. Gross J. J. (2014). Self-control and grit: related but separable determinants of success. Curr. Dir. Psychol. Sci. 23, 319–325. doi: 10.1177/0963721414541462, PMID: 26855479 PMC4737958

[ref19] Duckworth A. L. Peterson C. Matthews M. D. Kelly D. R. (2007). Grit: perseverance and passion for long-term goals. J. Pers. Soc. Psychol. 92, 1087–1101. doi: 10.1037/0022-3514.92.6.1087, PMID: 17547490

[ref20] Duckworth A. L. Quinn P. D. (2009). Development and validation of the short grit scale (grit-s). J. Pers. Assess. 91, 166–174. doi: 10.1080/00223890802634290, PMID: 19205937

[ref21] Dunston E. R. Messina E. S. Coelho A. J. Chriest S. N. Waldrip M. P. Vahk A.. (2022). Physical activity is associated with grit and resilience in college students: is intensity the key to success? J. Am. Coll. Heal. 70, 216–222. doi: 10.1080/07448481.2020.1740229, PMID: 32240056

[ref22] Eisenberg D. Gollust S. E. Golberstein E. Hefner J. L. (2009). The role of mental health in the college dropout process. J. Health Soc. Behav. 50, 459–477.

[ref23] Eskreis-Winkler L. Shulman E. P. Duckworth A. L. (2014). Survivor mission: do those who survive have a drive to thrive at work? J. Posit. Psychol. 9, 209–218. doi: 10.1080/17439760.2014.888579, PMID: 24748898 PMC3987915

[ref24] Fitzgerald C. McEwan D. (2020). Exploring the relationship between physical activity and grit in university students: a longitudinal study. J. Sport Behav. 43, 325–341.

[ref25] Fletcher T. Hodge K. Mallett C. (2020). The role of coaches in the development of young athletes: a review of the literature. Int. J. Sports Sci. Coaching 15, 341–352.

[ref26] Gerovasili V. Agaku I. T. Vardavas C. I. Filippidis F. T. (2015). Levels of physical activity among adults 18-64 years old in 28 European countries. Prev. Med. 81, 87–91. doi: 10.1016/j.ypmed.2015.08.005, PMID: 26299619

[ref27] Gilchrist J. D. Fong A. J. Herbison J. D. Sabiston C. M. (2017). Feelings of pride are associated with grit in student-athletes and recreational runners. Psychol. Sport Exerc. 7. doi: 10.1016/j.psychsport.2017.12.009

[ref28] Habelt L. Kemmler G. Defrancesco M. Spanier B. Henningsen P. Halle M.. (2023). Why do we climb mountains? An exploration of features of behavioural addiction in mountaineering and the association with stress-related psychiatric disorders. Eur. Arch. Psychiatry Clin. Neurosci. 273, 639–647. doi: 10.1007/s00406-022-01476-8, PMID: 35980451 PMC10085896

[ref29] Hattie J. Marsh H. W. Neill J. T. Richards G. (1997). Adventure education and outward bound: out-of-class experiences that make a lasting difference. Rev. Educ. Res. 67, 43–87. doi: 10.2307/1170619

[ref30] Hidi S. Renninger K. A. (2006). The four-phase model of interest development. Educ. Psychol. 41, 111–127. doi: 10.1207/s15326985ep4102_4

[ref31] Hill P. L. Burrow A. L. Bronk K. C. (2016). Persevering with positivity and purpose: an examination of purpose commitment and positive affect as predictors of grit. J. Happiness Stud. 17, 257–269. doi: 10.1007/s10902-014-9593-5

[ref32] Jordan M. H. Gabriel T. J. Russell T. Walker W. J. Schraeder M. (2015). An integrative approach to identifying factors related to long-term career commitments. Career Dev. Int. 20, 163–178. doi: 10.1108/CDI-05-2013-0071

[ref33] Lam K. K. L. Zhou M. (2019). Examining the relationship between grit and academic achievement within K-12 and higher education: a systematic review. Psychol. Sch. 56, 1654–1686. doi: 10.1002/pits.2230281

[ref34] Lee D. H. Reasoner K. Lee D. (2023). Grit: what is it and why does it matter in medicine? Postgrad. Med. J. 99, 535–541. doi: 10.1136/postgradmedj-2021-140806, PMID: 37319151

[ref35] Li L. Chu Z. Li F. Li J. Wang K. Zhou Y. (2025). The effect of mountaineering on the grit of college students: an empirical study. PeerJ 13:e19086. doi: 10.7717/peerj.19086, PMID: 40093403 PMC11910154

[ref36] Martin J. Toczko M. Locke E. McCarthy R. Milani I. Barrios N.. (2023). Influence of grit on physical activity, sitting time and dietary behaviors: a multi-study analysis. Sustainability. 15:211. doi: 10.3390/su15010211

[ref37] Meriac J. P. Slifka J. S. LaBat L. R. (2015). Work ethic and grit: an examination of empirical redundancy. Pers. Individ. Differ. 86, 401–405. doi: 10.1016/j.paid.2015.07.009

[ref38] Mian M. (2018). Research on the application of student autonomous interactive teaching mode in college physical education curriculum teaching. Sports Trend. 11:2.

[ref39] Nan Z. (2018). Research on the influencing factors of college students' grit. Nanchang: Nanchang University.

[ref40] Nothnagle E. A. Knoester C. (2022). Sport participation and the development of grit. Leis. Sci. 47, 1–18. doi: 10.1080/01490400.2022.2090037

[ref41] Parker L. (2021). Meeting diverse needs in physical education: strategies for inclusion. J. Physical Educ. Recreation Dance 92, 50–56.

[ref42] Patall E. A. Cooper H. Robinson J. C. (2008). The effects of choice on intrinsic motivation and related outcomes: a meta-analysis of research findings. Psychol. Bull. 134, 270–300. doi: 10.1037/0033-2909.134.2.270, PMID: 18298272

[ref43] Razali N. M. Wah Y. B. (2011). Power comparisons of Shapiro-wilk, Kolmogorov-Smirnov, Lilliefors and Anderson-Darling tests. J. Statis. Model. Anal. 2, 21–33.

[ref44] Roberta F. Hermundur S. Raul A. Ana F. S. Ricardo L. Filipe M. C. (2021). Passion, grit, and mindset in undergraduate sport sciences students. New Ideas Psychol. 62. doi: 10.1016/j.newideapsych.2021.100870

[ref45] Robertson-Kraft C. Duckworth A. L. (2014). True grit: trait-level perseverance and passion for long-term goals predicts effectiveness and retention among novice teachers. Teachers College Rec. 116, 1–27. PMC4211426 25364065

[ref46] Rutberg S. Nyberg L. Castelli D. Lindqvist A. K. (2020). Grit as perseverance in physical activity participation. Int. J. Environ. Res. Public Health 17:807. doi: 10.3390/ijerph17030807, PMID: 32012973 PMC7037268

[ref47] Ryan R. M. Deci E. L. (2000). Self-determination theory and the facilitation of intrinsic motivation, social development, and well-being. Am. Psychol. 55, 68–78. doi: 10.1037//0003-066x.55.1.68, PMID: 11392867

[ref48] Schimschal S. E. Visentin D. Kornhaber R. Cleary M. (2021). Grit: a concept analysis. Issues Ment. Health Nurs. 42, 495–505. doi: 10.1080/01612840.2020.1814913, PMID: 32915678

[ref49] Schunk D. H. Pintrich P. R. Meece J. L. (2008). Motivation in education: Theory, research, and applications. Routledge: Pearson Education.

[ref50] Smith R. Johnson A. (2019). The impact of traditional sports on student motivation and performance. Phys. Educ. Sport Pedagog. 24, 275–290.

[ref51] Tucker-Drob E. M. Briley D. A. Engelhardt L. E. Mann F. D. Harden K. P. (2016). Genetically-mediated associations between measures of childhood character and academic achievement. J. Pers. Soc. Psychol. 111, 790–815. doi: 10.1037/pspp0000098, PMID: 27337136 PMC5073013

[ref52] Wen-Ge P. (2007). Study on outdoor education and curricular design in american higher education: Bulletin of Sport Science & Technology.

[ref53] Williams M. Roberts J. Green T. (2020). The benefits of outdoor learning: a systematic review. Int. J. Environ. Sci. Educ. 15, 567–582.

[ref54] Xiao S. (2019). Investigation and analysis of the influence of physical education curriculum content on college students' interest in physical education. Contemporary Sports Technol. 7:2.

[ref55] Yeager D. S. Romero C. Paunesku D. Hulleman C. S. Schneider B. Hinojosa C.. (2016). Using design thinking to improve psychological interventions: the case of the growth mindset during the transition to high school. J. Educ. Psychol. 108, 374–391. doi: 10.1037/edu0000098, PMID: 27524832 PMC4981081

[ref56] Yu H. Zhu T. Tian J. Zhang G. Wang P. Chen J.. (2024). Physical activity and self-efficacy in college students: the mediating role of grit and the moderating role of gender. PeerJ 12:e17422. doi: 10.7717/peerj.17422, PMID: 38803579 PMC11129692

